# Self-condensation-assisted chemical vapour deposition growth of atomically two-dimensional MOF single-crystals

**DOI:** 10.1038/s41467-024-48050-5

**Published:** 2024-04-29

**Authors:** Lingxin Luo, Lingxiang Hou, Xueping Cui, Pengxin Zhan, Ping He, Chuying Dai, Ruian Li, Jichen Dong, Ye Zou, Guoming Liu, Yanpeng Liu, Jian Zheng

**Affiliations:** 1 Beijing National Laboratory for Molecular Sciences, Key Laboratory of Organic Solids, Institute of Chemistry, Chinese Academy of Sciences, 100190 Beijing, China; 2 University of Chinese Academy of Sciences, 100049 Beijing, China; 3 State Key Laboratory of Mechanics and Control for Aerospace Structures and Institute for Frontier Science, Nanjing University of Aeronautics and Astronautics, 210016 Nanjing, China

**Keywords:** Two-dimensional materials, Metal-organic frameworks

## Abstract

Two-dimensional metal-organic frameworks (MOFs) have a wide variety of applications in molecular separation and other emerging technologies, including atomically thin electronics. However, due to the inherent fragility and strong interlayer interactions, high-quality MOF crystals of atomic thickness, especially isolated MOF crystal monolayers, have not been easy to prepare. Here, we report the self-condensation-assisted chemical vapour deposition growth of atomically thin MOF single-crystals, yielding monolayer single-crystals of poly[Fe(benzimidazole)_2_] up to 62 μm in grain sizes. By using transmission electron microscopy and high-resolution atomic force microscopy, high crystallinity and atomic-scale single-crystal structure are verified in the atomically MOF flakes. Moreover, integrating such MOFs with MoS_2_ to construct ultrathin van der Waals heterostructures is achieved by direct growth of atomically MOF single-crystals onto monolayer MoS_2_, and enables a highly selective ammonia sensing. These demonstrations signify the great potential of the method in facilitating the development of the fabrication and application of atomically thin MOF crystals.

## Abstract (web summary)

2D MOFs have a variety of applications, including molecular separation and atomically thin electronics. Here authors demonstrate the preparation of monolayer and few-layer MOF single-crystals using a self-condensation-assisted CVD method.

## Introduction

Owing to the quantum confinement in two dimensions, single-layered crystals exhibit many exotic physical and chemical properties that differ from their bulk counterparts, thus triggering an explosion in the search for atomically thin materials^1–8^. Recently, two-dimensional (2D) metal-organic frameworks (MOFs) with inherent molecular pore/cavity structures that consist of metal ions connected by organic ligands have emerged as one increasingly compelling field due to diverse designable structures and tunable properties^9–13^. At the atomic thickness, 2D MOFs feature rapid mass transfer^11^, exceptional carrier transport^13,14^ and an extremely high proportion of exposed active sites^15,16^, and have easily identifiable atomic structures and bonding arrangements for an ideal model to search for precise structure-performance relationship^17^. Because of these unique characteristics, 2D MOFs have shown great potential as building blocks for significant technologies in applications such as molecular sensing^18^, gas separation^11,19^, catalysis^15–17^ and superconductor^20^. A key towards the fulfilment of these features and notable applications is to reliably prepare atomically thin MOF crystals in high quality. Great effort has thus been devoted to the development of their preparation methods, such as micromechanical exfoliation^10^, liquid exfoliation^16,21^, and wet-chemical method^14,17^. However, due to the inherent fragility and strong interlayer interactions in the bulk crystals, 2D MOFs obtained by the above methods are usually thick or have a few layers with small size and/or limited crystallinity. It is highly desired to reliably prepare high-quality and large MOF crystals with atomic thickness, especially the isolated monolayers.

Chemical vapour deposition (CVD), a typical bottom-up method, is considered to be a robust way to grow 2D materials with controllable thickness, scalable size and high crystal quality. A range of high-quality monolayer 2D materials, such as graphene^22^, *h*-BN^6,23^, and transition metal dichalcogenides (TMDs)^24–26^, have been prepared by the CVD method. Therefore, it is promising to prepare high-quality monolayer MOFs with large sizes through the CVD technique. Recently, a multistep CVD method has been demonstrated to achieve the growth of MOF thin films (such as ZIF-8)^27^, which marks notable progress in the CVD synthesis of MOFs. Nonetheless, several fundamental challenges remain in terms of CVD growth of atomically thin MOF single-crystals. In a typical CVD process for growing inorganic nanomaterials and graphene, atoms can diffuse rapidly over a long distance on the substrate surface at high growth temperatures (≥700 °C), resulting in the generation of large monolayer domains. While in the CVD growth of MOFs, a low growth temperature (typically below 300 °C) and strong interactions between ligand and substrate, as well as the large mass and volume of ligand molecules, together cause the ligand precursor molecules to have a short free path on the substrate surface. Dense nucleation and growth of small grains of tens of nanometres often ensue, resulting in thick polycrystalline films of MOFs. Thus far, the growth of large, single-crystal, atomically thin MOFs, especially for the monolayers, has yet to be realized using the CVD method.

Here, we demonstrate a self-condensation-assisted CVD (SCA-CVD) growth of atomically thin, single-crystal MOF, in which monolayer poly[Fe(benzimidazole)_2_] single-crystals were obtained with grain sizes up to 62 μm. The self-condensation of the precursor induced by a temperature gradient design during the CVD process is supposed to be critical in mediating the growth of atomically thin, large-sized MOF single crystals. Characterization by transmission electron microscopy (TEM) and high-resolution atomic force microscopy (HRAFM) have revealed the high crystallinity and atomic structures of single-crystals in the monolayer and few-layer MOF flakes. In addition, good compatibility of the SCA-CVD growth of MOF crystals has been shown and enabled atomically thin MOF single-crystals to grow directly onto monolayer MoS_2_ to create an ultrathin van der Waals (vdW) heterostructure of MOF/MoS_2_. Integration combining the precise gate effect of MOF crystal and the high sensitivity of monolayer MoS_2_ is testified in such heterostructure to engender highly selective ammonia sensing.

## Results

### SCA-CVD growth and characterization of atomically thin MOF single-crystals

**Fig. 1:**
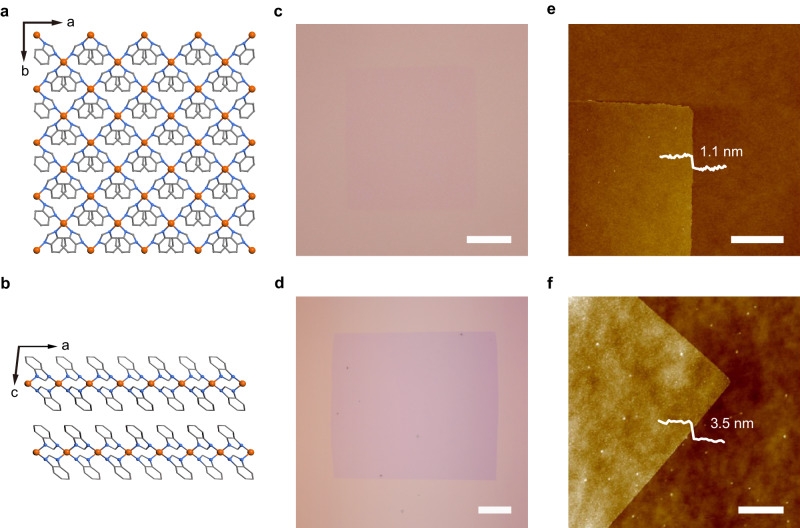
Atomically thin MOF single crystals of Fe_n_(bim)_2n_. **a** Crystal structure of a Fe_n_(bim)_2n_ single crystal viewed down the *c*-axis (*a*–*b* plane), i.e., the lattice structure of a single layer of Fe_n_(bim)_2n_, where the iron atoms are represented in orange, nitrogen atoms in blue, carbon atoms in grey and hydrogen atoms are omitted for clarity^10^. **b** Layered crystal structure of a Fe_n_(bim)_2n_ single crystal viewed along the *b*-axis^10^. **c**, **d** Optical images of a typical monolayer (**c**) and three-layered (**d**) Fe_n_(bim)_2n_ single crystal grown on a SiO_2_/Si substrate by SCA-CVD. **e**, **f** Corresponding AFM images of the Fe_n_(bim)_2n_ single crystal in (**c**) and (**d**). Scale bars, 10 μm in (**c**), 20 μm in (**d**) and 1 μm in (**e**, **f**).

The poly[Fe(benzimidazole)_2_], denoted as Fe_n_(bim)_2n_, is a typical Van der Waals layered MOF. A structural illustration (Fig. 1a, b) of a single-crystal MOF of Fe_n_(bim)_2n_ shows a layered construction in which distorted tetrahedral Fe(II) centres are linked with bridging bis-monodentate benzimidazole ligands in the *a*–*b* plane^10^. We conducted the SCA-CVD growth of the atomically thin layer of Fe_n_(bim)_2n_ on SiO_2_/Si substrates using powdered ferrocene and benzimidazole as precursors in a two-zone tube furnace system (see the “Methods” section for details). Monolayer and few-layer Fe_n_(bim)_2n_ crystals were obtained and first identified under an optical microscope. As shown in Fig. 1c, d and Supplementary Fig. 1, a well-defined rectangle shape was clearly exhibited by the isolated flakes, which is well aligned with the coordination framework symmetry of Fe_n_(bim)_2n_ single-crystals. The exact thicknesses of the flakes were measured to be 1.1 and 3.5 nm (Fig. 1f, g), corresponding to monolayer and three-layer thicknesses, respectively. Statistics based on the AFM measurement showed that the ratio of the monolayer was ~50%. In addition, the Fe_n_(bim)_2n_ single-crystals grown by the SCA-CVD method were of considerable lateral dimensions, with lengths for the monolayer and the few-layer up to 62 and 105 μm, respectively. To the best of our knowledge, this was the first time monolayer MOF crystals were obtained with the CVD method, and the MOF crystal was also the largest monolayer single-crystal MOF obtained thus far. Furthermore, the SCA-CVD method allowed atomically thin single-crystal Fe_n_(bim)_2n_ to be grown on substrates, including KBr crystals, mica, sapphire and so on, where grown crystals showed a similar appearance to that grown on the SiO_2_/Si substrates (Supplementary Fig. 2). Also, atomically thin MOF crystals, including poly[Fe(5-methylbenzimidazole)_2_], poly[Fe(5-bromobenzimidazole)_2_], poly[Fe(5-chlorobenzimidazole)_2_] and poly[Zn(benzimidazole)_2_] were successfully prepared via SCA-CVD (Supplementary Figs. 3 and 4).

**Fig. 2:**
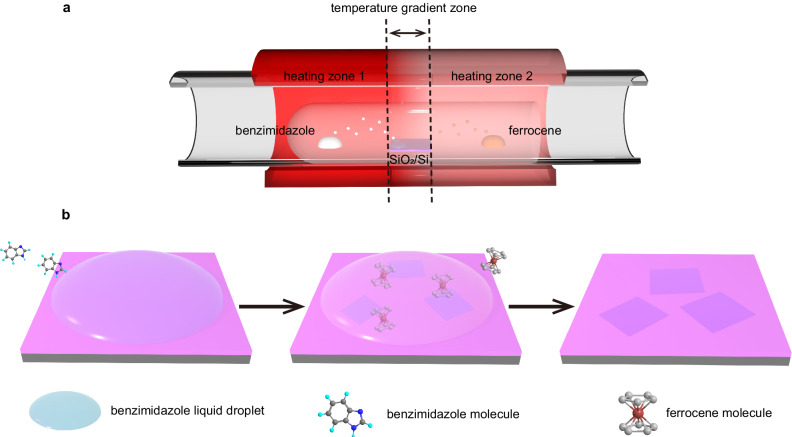
Self-condensation-assisted CVD (SCA-CVD) growth of atomically thin single crystals of Fe_n_(bim)_2n_. **a** Schematics of the set-up for SCA-CVD growth. **b** The proposed process of SCA-CVD growth involves liquid droplet formation due to the self-condensation of benzimidazole precursor vapour, dissolution and diffusion of the vapourized ferrocene molecules in the liquid droplets, formation of atomically thin single crystals of Fe_n_(bim)_2n_.

For SCA-CVD growth of MOFs, ferrocene and various benzimidazole derivatives can be easily obtained in large quantities, facilitating the extension of prepared MOF types, and they are easy to sublimate. The benzimidazole derivatives also demonstrate a suitable melting point in which a temporary liquid environment can be offered. Figure 2 schematically shows SCA-CVD growth. Self-condensation of the precursor into liquid was found to be induced through a temperature gradient design (Fig. 2a) in the CVD process. As shown in Supplementary Fig. 5, obvious vapour-condensed liquid droplets of benzimidazole appeared on the surface of the growth substrate in the gradient zone and on the inner wall of the nearby quartz tube upon thermal sublimation of the precursor. Temperature monitoring (Supplementary Fig. 6) also showed that the substrate temperatures (~200 °C) during the growth stage were higher than the melting point (169–171 °C) of benzimidazole, indicating that the benzimidazole deposited on the substrate existed in liquid state at the growth stage. To further investigate the growth process, a control experiment was carried out. The self-condensed liquid droplets at different growth times were extracted from the same area of the growth substrate and analysed by gas chromatography‒mass spectrometry (GC‒MS) (Supplementary Fig. 7). A time-dependent evolution of components in the liquid droplets was revealed, in which the droplets gradually changed from pure benzimidazole into a mixture of benzimidazole and ferrocene. These experimental results depicted a probable process of SCA-CVD growth (Fig. 2b). First, benzimidazole precursor was sublimated into vapour, diffused to the substrate, and then condensed into liquid upon a negative temperature gradient. The vapourized ferrocene subsequently dissolved and diffused in the benzimidazole droplets, accompanied by coordination reactions with benzimidazole molecules. Lastly, the Fe_n_(bim)_2n_ nucleated and grew into crystals on the substrate. It should be noted that the involved liquid droplets in the SCA-CVD growth are formed upon vapourization to condensation, and thus they are of high purity and can be fully removed from the growing crystals by a simple vacuum treatment due to the volatility of the precursors without leaving impurities, which is completely different from ordinary solvents and in situ formed liquids.

**Fig. 3:**
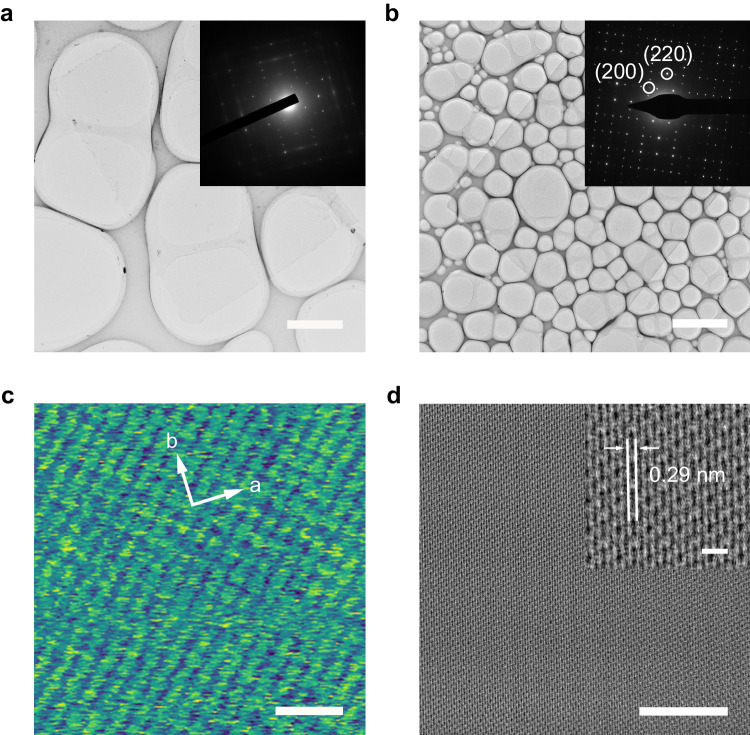
Crystal structure of atomically thin single crystals of Fe_n_(bim)_2n_. **a** Low-magnification TEM image of a typical monolayer Fe_n_(bim)_2n_ single crystal. The inset shows the corresponding SAED pattern in (**a**). **b** Low-magnification TEM image of a typical few-layer Fe_n_(bim)_2n_ single crystal, with its SAED pattern shown in the inset. **c** HRAFM image of a typical monolayer Fe_n_(bim)_2n_ single crystal. **d** Cryogenic TEM image of a typical few-layer Fe_n_(bim)_2n_ single crystal. The inset shows the higher-magnification image, corresponding to the (002) plane of the Fe_n_(bim)_2n_ crystal. The cryogenic TEM was conducted under a low dose of 17.56 e/Å^2^/s. Scale bars, 2 μm in (**a**), 5 μm in (**b**), 2 nm in (**c**), 10 nm in (**d**) and 1 nm in inset of (**d**).

To examine the crystal quality and structure of the SCA-CVD-grown Fe_n_(bim)_2n_ flakes, TEM, selective area electron diffraction (SAED), and HRAFM characterizations were performed. Fe_n_(bim)_2n_ flakes grown on KBr crystals were employed for TEM observation, as the excellent water solubility of KBr crystals allows Fe_n_(bim)_2n_ flakes to be gently transferred to TEM grids in water, thus preserving intrinsic properties of the transferred flakes. As shown in Fig. 3a, b, monolayer and few-layer Fe_n_(bim)_2n_ flakes exhibit typical rectangular shapes with clean and uniform surfaces, which are consistent with the observations obtained by the optical microscope. Collected along the [001] axis, SAED patterns of these flakes (inset of Fig. 3a, b) show clear Bragg diffraction signals and only one set of quasi-fourfold symmetry diffraction spots, which is in good agreement with the simulated electron diffraction pattern (Supplementary Fig. 8), suggesting that both the single-layer and few-layer Fe_n_(bim)_2n_ flakes are single-crystals with high crystallinity. An individual Fe_n_(bim)_2n_ flake in a rectangle shape has been further confirmed as a single crystal by collecting SAED patterns at different positions throughout the entire flake (Supplementary Figs. 9–11). A regular periodic lattice structure is displayed in the few-layer Fe_n_(bim)_2n_ flake, as shown in the cryogenic TEM image (Fig. 3d). The lattice spacing was measured to be ~2.9 Å, close to the *d*_220_ of the Fe_n_(bim)_2n_ crystal, which corresponds to (002) plane of the Fe_n_(bim)_2n_ crystal. Almost no point defects and voids were initially observed within the entire field of view, further confirming high crystallinity of the Fe_n_(bim)_2n_ flake. As monolayer MOFs suffer visible and rapid disordering under electron beam irradiation, which is common for 2D MOF crystal characterization with high-resolution TEM, we examined the atomic structure of monolayer Fe_n_(bim)_2n_ with HRAFM. As shown in Fig. 3c and Supplementary Fig. 12, rectangular Fe_n_(bim)_2n_ units interconnecting into a periodically arranged network with an internal angle of about 90° are clearly observed in the monolayer. The lattice constants of *a* and *b* were measured to be 8.3 and 8.4 Å, respectively, which matched well with the crystallographic data, suggesting good quality of the grown monolayer Fe_n_(bim)_2n_. The elemental information and chemical bonding of Fe_n_(bim)_2n_ were also confirmed by scanning transmission electron microscopy (STEM) energy-dispersive X-ray spectroscopy (EDS) mapping images (Supplementary Fig. 13), X-ray photoelectron spectroscopy (XPS, Supplementary Fig. 14) and Fourier transform infra-red (FT-IR, Supplementary Fig. 15).

### Mechanism of SCA-CVD growth of single-crystal 2D MOFs

Compared with previous studies, the SCA-CVD method demonstrates the following advantages: it has achieved the growth of large-sized single-layer MOFs and a relatively high single-layer ratio by simple one-step operations, and the grown 2D MOFs single crystals are clean and of good crystallinity. The involvement of the self-condensation liquid in the CVD process is supposed to play a vital role in enabling the growth of the atomically thin large-sized Fe_n_(bim)_2n_ single-crystals. During the traditional CVD process for 2D MOF crystals growing, the coordination of precursors in the gas phase is difficult without catalysts; the low growth temperature typically leads to irreversible crystal growth, resulting in products with poor crystallinity, and kinetic limitations caused by small diffusion rates and radii of the localized ligand precursors also tend to generate small grains. In the SCA-CVD method for MOF growth, a pure temporary liquid environment is introduced into the growth system through the self-condensation of the precursor. This liquid environment serves as a buffer layer to reduce disturbance inside and outside the liquid on the crystal nucleation and growth and allows precursor molecules to be uniformly and stably distributed within the liquid. An increased diffusion rate and radius of the precursor molecules compared with that on the gas–solid interface can be achieved in the liquid to lessen the kinetic limitations of large grain growth along the lateral direction. In addition, similar to most 2D materials, the atoms in a Fe_n_(bim)_2n_ layer interact through strong chemical bonds, while the interactions between layers are weak van der Waals force^10^. Previous density functional theory (DFT) studies have shown that the layer−layer interactions in most van der Waals materials are around 0.03–0.2 eV per atom, while the strength of typical chemical bonds is between 2 and 8 eV per atom^28^. The strength of intralayer interaction is 1–2 orders of magnitude higher than that of interlayer interaction. Because of the stronger intralayer binding, attaching atoms to an edge of a Fe_n_(bim)_2n_ layer to facilitate the in-plane extension is much easier than adsorbing atoms on the surface of a Fe_n_(bim)_2n_ layer for the growth of multilayers. More specifically, the nucleation energy for a new layer is the maximum of $\alpha \sqrt{A}-\beta A$, where $A$ is the nucleus size, and the first and second terms represent the positive and negative contributions from the edge and bulk of the nucleus, respectively^29^. In comparison, the nucleation energy for the growth of an edge (in-plane extension) is the energy of new dangling bonds when forming a unitcell of Fe_n_(bim)_2n_ on the edge^30^. Under a near equilibrium growth condition (low *β*), the critical nucleation size for a new layer will be much larger than a unitcell of Fe_n_(bim)_2n_, and more dangling bonds involves in the formation of a new layer, which results in a higher nucleation energy for a new layer. Therefore, selective in-plane growth of Fe_n_(bim)_2n_ is favourable in an equilibrium reversible thermodynamic process, as the case in CVD growth of most 2D Van der Waals crystals. Notably, the liquid nature allows dynamic bonding between the metal and ligand nodes of MOFs, in which the metal−ligand bonds formed, broke and reformed to correct disorder or premature structure termination, which is crucial in forming a crystalline and ordered structure^31^. In addition, as the separate nuclei in the liquid grow and approach each other, due to the preference of the free energy of the system to decrease, interface elimination processes prefer to occur to reduce the total area of the grain boundaries^32^. It energetically favoured the formation of large-sized, single-crystalline MOF crystals in the liquid environment. These ultimately afford the growth of large single-crystal 2D MOFs.

### Selective ammonia sensing of ultrathin vdW heterostructure of Fe_n_(bim)_2n_/MoS_2_

**Fig. 4:**
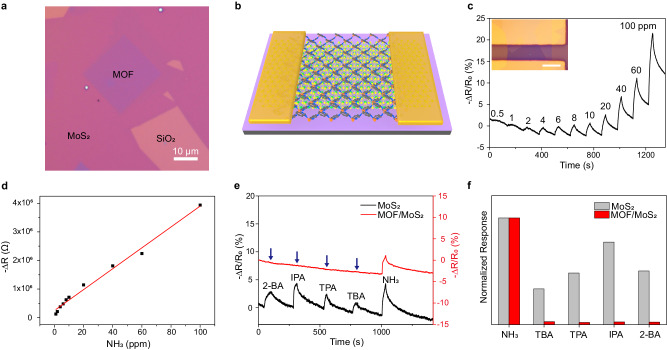
Ultrathin van der Waals (vdW) heterostructure of Fe_n_(bim)_2n_/MoS_2_. **a** Optical image of an ultrathin vdW heterostructure of Fe_n_(bim)_2n_/MoS_2_. **b** Schematic illustration of a device based on the vdW heterostructure of Fe_n_(bim)_2n_/MoS_2_, in which the periodic network of Fe_n_(bim)_2n_ represented by the dark grey grid is superimposed on a monolayer MoS_2_ denoted as atomic structure. **c** Real-time sensing behaviour of a Fe_n_(bim)_2n_/MoS_2_ sensor upon consequent NH_3_ exposures at various concentrations. The inset shows an optical image of the Fe_n_(bim)_2n_/MoS_2_ sensor. ∆*R* is the change in sensor resistance, defined as *R*_Sensor_−*R*_0_. Scale bar, 20 μm. **d** Plot of resistance change (black solid squares) of Fe_n_(bim)_2n_/MoS_2_ sensor as a function of NH_3_ concentration. The red line indicates the fitted line. **e** Sensing response of a monolayer MoS_2_ (black line) and a Fe_n_(bim)_2n_/MoS_2_ (red line) when exposed to NH_3_ gas, tert-butylamine (TBA) vapour, tert-pentylamine (TPA) vapour, isopropylamine (IPA) vapour and 2-butylamine (2-BA) vapour of the same concentration (20 ppm). The blue arrow represents the injection of analytes. **f** Normalized sensing response of the monolayer MoS_2_ and the Fe_n_(bim)_2n_/MoS_2_ heterostructure in (**e**).  **c**–**f** are provided as a Source Data file.

The demonstrations of high-quality growth of atomically thin Fe_n_(bim)_2n_ flakes make it desirable to explore their integration into devices. Ultrathin vdW heterostructures constitute a simple but powerful platform for studying fundamental physics and creating functional devices in the 2D limit, in which combining or extending properties are accessible through the synergy of their constituent materials^33^. Since the SCA-CVD method is general to a variety of substrates, we fabricated a 2D vdW heterostructure composed of atomically thin Fe_n_(bim)_2n_ single-crystal directly grown onto MoS_2_ monolayers via the SCA-CVD method in which the transfer processes that often cause quality damage and contamination are not involved. (Fig. 4 and Supplementary Fig. 16; see the “Methods” section for details). No obvious interaction between Fe_n_(bim)_2n_ and MoS_2_ was observed in the heterostructure (Supplementary Fig. 17). Monolayer MoS_2_ is characterized by a high sensitivity response due to its high surface-to-volume ratio and excellent semiconducting properties. To evaluate such vdW heterostructures, gas-sensing measurements were performed. The large size of SCA-CVD grown Fe_n_(bim)_2n_ single crystals enables the direct construction of electrodes for the heterostructure sensors using a hollow mask method without the use of electron beam lithography technology (see the “Methods” section). Figure 4c shows a typical sensing response (*S*) of a Fe_n_(bim)_2n_/MoS_2_ sensor (Supplementary Fig. 18) to varied concentrations of NH_3_ gas, where *S* is defined as (*R*_Sensor_−*R*_0_)/*R*_0_, *R*_0_ and *R*_Sensor_ are the resistance of the sensor before and after gas introduction. The sensor responded almost immediately to NH_3_ exposure, given that the response is limited by the opening speed of the solenoid valve and the gas mixing speed in the sensing chamber. Clear electrical resistance decreases were observed in the sensors upon NH_3_ exposure, even at sub-ppm levels (500 ppb). The sensitivity upon NH_3_ exposure is found to be comparable to that of independent 2D MoS_2_ sensors^34^, which suggests the good sensitivity of the Fe_n_(bim)_2n_/MoS_2_. In addition, good linear sensor sensitivity can be achieved when the NH_3_ concentration ranges from 1 to 100 ppm (Fig. 4d), indicating a feasible determination of gas concentration.

Independent monolayer MoS_2_ is highly sensitive to environmental active stimuli, as their atoms are almost completely exposed; however, it is theoretically impossible to distinguish interfering substances with similar chemical properties by monolayer MoS_2_. Amine gases/vapours such as NH_3_, tert-butylamine (TBA), tert-pentylamine (TPA), isopropylamine (IPA) and 2-butylamine (2-BA) are electron donors; When expose them to a monolayer MoS_2_, the absorbed molecules on the surface of MoS_2_ would shift the Fermi level to the conduction band, leading to electrical resistance decreases of MoS_2_^33^. As shown in Fig. 4e, obvious electrical resistance decreases were all observed in a monolayer MoS_2_ sensor to exposure of NH_3_, TBA, TPA, IPA and 2-BA, respectively, with *S* of −5.45% for NH_3_, −1.82% for TBA, −2.63% for TPA, −4.21% for IPA and −2.74% for 2-BA (at the same concentration). However, when exposing the above analytes to sensors fabricated with the Fe_n_(bim)_2n_/MoS_2_ heterostructure, a rapid drop in device resistance only occurs as gaseous NH_3_ is injected, and almost no change in the resistance is observed with an injection of other amine vapours. The *S* of the Fe_n_(bim)_2n_/MoS_2_ sensor for the same concentrations of NH_3_, TBA, TPA, IPA and 2-BA are about −4.14%, −0.11%, −0.08%, −0.10% and −0.09%, respectively. Calculating selectivity coefficient of the NH_3_ ($D_{{\rm NH}_{3}}$) that is defined as the ratio of responses of NH_3_ and another interfered gas/vapour, that is $D_{{\rm NH}_{3}}$ = $S_{{\rm NH}_{3}}$/*S*_i_, there is at least one order of magnitude increase in $D_{{\rm NH}_{3}}$ for the Fe_n_(bim)_2n_/MoS_2_ (Fig. 4f), where $D_{{\rm NH}_{3}}$ was 2.99 to TBA, 2.07 to TPA, 1.29 to IPA and 1.99 to 2-BA for the monolayer MoS_2_ sensor, and 36.95 to TBA, 50.19 to TPA, 40.18 to IPA and 43.32 to 2-BA for the Fe_n_(bim)_2n_/MoS_2_ sensor. This proves a clearly enhanced NH_3_ selective response of the Fe_n_(bim)_2n_/MoS_2_ heterostructure among amine vapours/gases. In addition, the NH_3_ selectivity of the Fe_n_(bim)_2n_/MoS_2_ sensor has good reproducibility (Supplementary Fig. 19).

The good sensitivity and selective ammonia response of the ultrathin vdW heterostructure of Fe_n_(bim)_2n_/MoS_2_ (Supplementary Table 1) reflect the integration of the high sensitivity of monolayer MoS_2_ and the precise gate effect of MOF crystals. The MOF crystal of Fe_n_(bim)_2n_ is known to be a microporous crystalline material with inherent size exclusion characteristics. According to the crystallographic data, the theoretical aperture size of Fe_n_(bim)_2n_ unit is estimated to be about 0.21 nm (Supplementary Fig. 20). Considering the structure flexibility of MOF owing to the aromatic rings flip-flop, the effective pore size should be slightly larger than the theoretical aperture size^11,35^. Organic amine vapours, including TBA, TPA, IPA and 2-BA, all have kinetic diameters far exceeding the effective aperture size of Fe_n_(bim)_2n_. As expected, due to the size exclusion effect by the small aperture size of Fe_n_(bim)_2n_, the gate effect of the Fe_n_(bim)_2n_ layer prevents these organic amine vapours from accessing the surface of MoS_2_, causing almost no gas sensing responses. While NH_3_ molecules with a kinetic diameter of ~2.6 Å, which is comparable to the effective pore aperture of Fe_n_(bim)_2n_, can easily pass through the Fe_n_(bim)_2n_ network and reach the MoS_2_ surface, resulting in gas responses. Notably, when epitaxially grown onto monolayer MoS_2_ forming a vdW heterostructure, the Fe_n_(bim)_2n_ crystals stacked compactly on the MoS_2_ with an atomically clean interface. In addition, the grown Fe_n_(bim)_2n_ crystals were intact single-crystals without grain boundary defects to generate undesired gas permeation pathways. These enable the MOF crystal to maximize its precise gate effect in the heterostructure, which is different from the case of previously reported MOF thin films. A strict nanochannel created by the Fe_n_(bim)_2n_ single-crystals is thus imposed on the monolayer MoS_2_, in which only molecules of a certain size are able to reach the MoS_2_. Besides, compared to the long nanopores in a MOF thin film, the Fe_n_(bim)_2n_ with atomic-level thickness presents ultra-short nanopores that allow the high sensitivity and fast response of MoS_2_ monolayers to be retained in the heterostructure, which is responsible for the measured good sensitivity of the Fe_n_(bim)_2n_/MoS_2_ to NH_3_ exposure.

In conclusion, our results demonstrate the preparation of high-quality MOF single-crystals with atomic thicknesses using a SCA-CVD method, in which large isolated monolayer single-crystals of Fe_n_(bim)_2n_ in high crystallinity were achieved. By directly growing atomically thin MOF single-crystals on monolayer MoS_2_ via the SCA-CVD method, we fabricated ultrathin vdW heterostructures of Fe_n_(bim)_2n_/MoS_2_, of which a highly selective ammonia response was shown to result from the synergy of MOF and MoS_2_. Our methodology inspires a synthetic pathway for creating high-quality atomically thin molecular frameworks that were previously inaccessible and can greatly promote their low-dimensional device integration and applications.

## Methods

### Growth of atomically thin Fe_n_(bim)_2n_ single crystals

The single-crystal Fe_n_(bim)_2n_ with atomic thickness was prepared by the SCA-CVD method at atmospheric pressure. Precursor powders, benzimidazole (Innochem, 99%, 0.15 mmol) and ferrocene (Adamas Beta, 99%, 0.15 mmol), were respectively loaded at two ends of a single-ended sealed quartz tube. Clean growth substrate, such as SiO_2_/Si and sapphire, was placed face up between the benzimidazole and ferrocene. The quartz tube containing precursors and growth substrate was then transferred into a two-zone furnace, with the benzimidazole and ferrocene, respectively, in the centres of the two heating zones and the growth substrate located in the temperature gradient zone. Before growth, the system was first degassed and then purged with ultra-purity nitrogen for 10 min at a flow rate of 200 sccm. The growth recipe was ramped from room temperature to 400 °C within 20 min for the heating zone (1, benzimidazole loading) while from room temperature to 150 °C for the heating zone (2, ferrocene loading) and sit 15 min at 400 and 150 °C. Ultra-purity nitrogen with 5 sccm was flowed in the whole growth process. After growth, the system was degassed to terminate the reaction and then opened the furnace for rapid cooling.

### GC-MS analysis

The liquid droplet components during the SCA-CVD process were analysed by gas chromatography-mass spectrometry GC–MS (SHIMADZU-QP2010) with a packed column DB-5 MS. Droplets located at the same position of the substrates were collected by breaking off the heating programme at desired times. A mixture of benzimidazole and ferrocene with a molar ratio of 1:1 was used as a reference sample for the GC–-MS test. All samples for GC–MS testing utilized ethanol as a solvent.

### Preparation of vdW 2D Fe_n_(bim)_2n_/MoS_2_ heterostructures

We first grew monolayer MoS_2_ crystals on a SiO_2_/Si substrate by CVD using a method reported in ref. ^24^. The as-grown MoS_2_ monolayers were transferred to the two-zone furnace as substrates for the growth of Fe_n_(bim)_2n_. The SCA-CVD growth of Fe_n_(bim)_2n_ was then conducted using the above similar recipes, in which atomically thin Fe_n_(bim)_2n_ crystals were directly grown on MoS_2_ monolayers. The vdW 2D Fe_n_(bim)_2n_/MoS_2_ heterostructures were thus fabricated.

### Device fabrication and measurements

The gas-sensing devices were fabricated through direct thermal evaporation deposition of 60 nm gold on top of the monolayer MoS_2_ flakes and Fe_n_(bim)_2n_/MoS_2_ heterostructures with a metal shadow mask. Gas-sensing experiments were carried out at room temperature in a gas-sensing chamber, where the devices were placed on a chip-carrier with electrically connected gold wires led out by a vacuum terminal block. The gas sensing chamber was maintained at a pressure of 100 Pa and balanced with pure nitrogen at a flow rate of 100 sccm. Analytes we used for the test were NH_3_ gas, isopropylamine vapour, 2-butylamine vapour, tert-butylamine vapour and tert-pentylamine vapour. Mix them with ultra-pure nitrogen in proportion to achieve desirable concentrations, and then inject them into the gas-sensing chamber by mass flow controllers with a flow rate of 100 sccm. Adjust the flow rate of pure nitrogen to zero when introducing the analyte to maintain a total constant flow rate of 100 sccm. The current of the devices during the gas-sensing test was monitored in real-time at a constant bias voltage (−1.0 V) using the two-probe with an Agilent B2902A Source Meter.

### Characterizations and simulation

Optical images were captured with a Nikon Eclipse LV 100D. AFM images were collected in tapping mode using a Bruker Dimension Icon Scanning Probe Microscope. HRAFM images were recorded on a Cypher VRS1250 in contact mode. The TEM experiments were carried out with a JEOL JEM-2100F operated at 200 kV. Cryogenic TEM image was obtained with a Thermo Scientific Themis 300 with a field-emission gun operating at 300 kV. Simulated SAED pattern was generated with SingleCrystal software.

### Supplementary information

Supplementary Information

Peer Review File

### Source data

Source Data

## Data Availability

The data that support the findings of this study are available from the corresponding authors upon request. The source data underlying Fig. 4 and Supplementary Figs. 6, 7, 14, 15, 17 and 19 are provided in the Source Data file. Source data are provided with this paper.
