# Supplementary material for: Self-condensation-assisted chemical vapour deposition growth of atomically two-dimensional MOF single-crystals
Source: Nat Commun. 2024 Apr 29;15:3618. doi: 10.1038/s41467-024-48050-5 (PMC11059375; doi:10.1038/s41467-024-48050-5)
Supplement: Supplementary file 1 — Supplementary Information [file 41467_2024_48050_MOESM1_ESM.pdf]

Supplementary Information

Self-condensation-assisted chemical vapour deposition growth of atomically two-dimensional MOF single-crystals

Lingxin Luo[1,2,†], Lingxiang Hou[1,†], Xueping Cui[1*], Pengxin Zhan[1,2], Ping He[1,2], Chuying Dai[1,2], Ruian Li[1], Jichen Dong[1], Ye Zou[1], Guoming Liu[1], Yanpeng Liu[3] and Jian Zheng[1*]

[1]Beijing National Laboratory for Molecular Sciences, Key Laboratory of Organic Solids, Institute of Chemistry, Chinese Academy of Sciences, Beijing 100190, China.
[2]University of Chinese Academy of Sciences, Beijing 100049, China.
[3]State Key Laboratory of Mechanics and Control for Aerospace Structures and Institute for Frontier Science, Nanjing University of Aeronautics and Astronautics, Nanjing, 210016, China.
[†]These authors contributed equally: Lingxin Luo, Lingxiang Hou.
*Corresponding author: zhengjian@iccac.ac.cn, cuixueping@iccas.ac.cn.

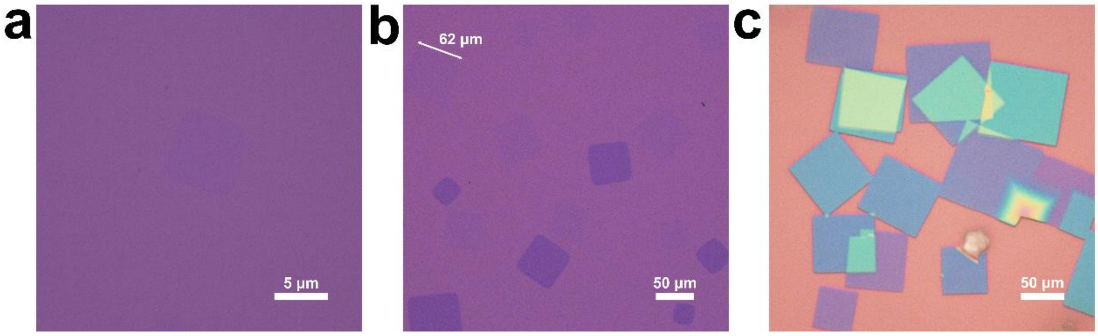

Supplementary Figure 1. Optical images of $Fe_n(bim)_{2n}$ flakes with growth time of 5 minutes (**a**), 15 minutes (**b**) and 40 minutes (**c**). It can be seen that the shorter the growth time, the smaller the average grain size of $Fe_n(bim)_{2n}$, and the sparser the grain distribution, and while the growth time extended, the average grain size of $Fe_n(bim)_{2n}$ increased, accompanied by an increase in thickness and distribution density.

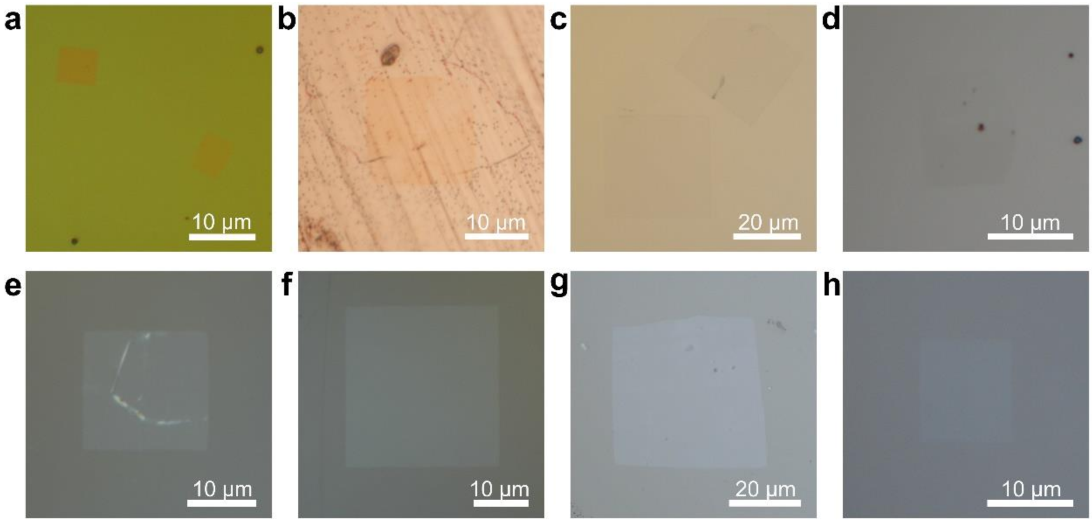

Supplementary Figure 2 | SCA-CVD growth of $Fe_n(bim)_{2n}$ grown on different substrates. Typical optical images of $Fe_n(bim)_{2n}$ flakes grown on SiN (**a**), Cu foil (**b**), sapphire (**c**), Si (**d**), quartz (**e**), KBr (**f**), glass (**g**) and mica (**h**).

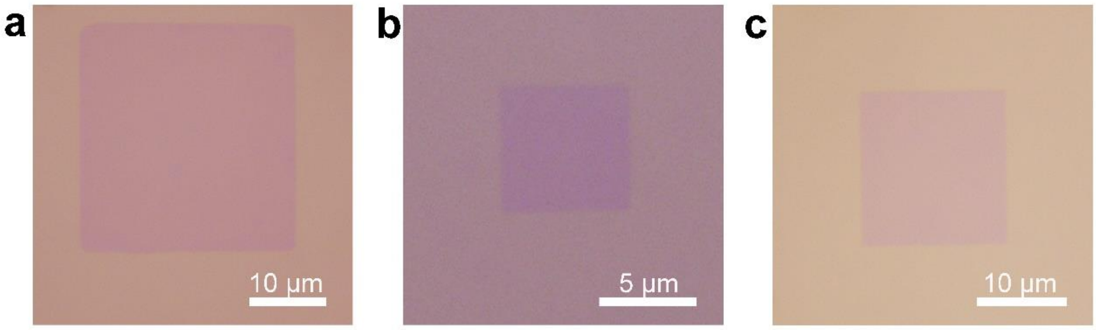

Supplementary Figure 3 | Generalization of SCA-CVD synthesis strategy to produce 2D MOF flakes. Optical images of the atomically thin MOF crystals grown by SCA-CVD method: poly[Fe(5-methylbenzimidazole)$_2$] (**a**), poly[Fe(5-chlorobenzimidazole)$_2$] (**b**) and poly[Fe(5-bromobenzimidazole)$_2$] (**c**). All the samples were grown on SiO_2/Si substrates.

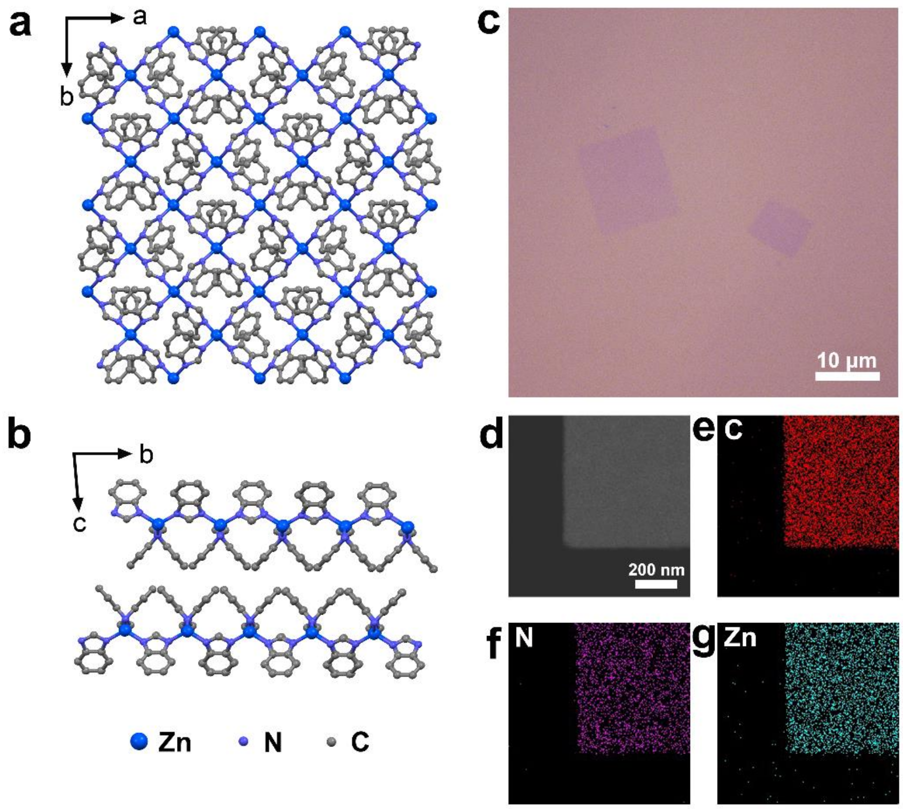

Supplementary Figure 4 | The SCA-CVD growth of poly[Zn(benzimidazole)$_2$] flakes. (**a**) Crystal structure of a poly[Zn(benzimidazole)$_2$] single crystal viewed down the *c* axis (*a*–*b* plane). (**b**) Layered crystal structure of a poly[Zn(benzimidazole)$_2$] single crystal. (**c**) Typical optical image of poly[Zn(benzimidazole)$_2$] flakes. (**d-g**) STEM-EDS mapping of poly[Zn(benzimidazole)$_2$] flake. The metal source is bis(2,2,6,6-tetramethyl-3,5-heptanedionato) zinc(II).

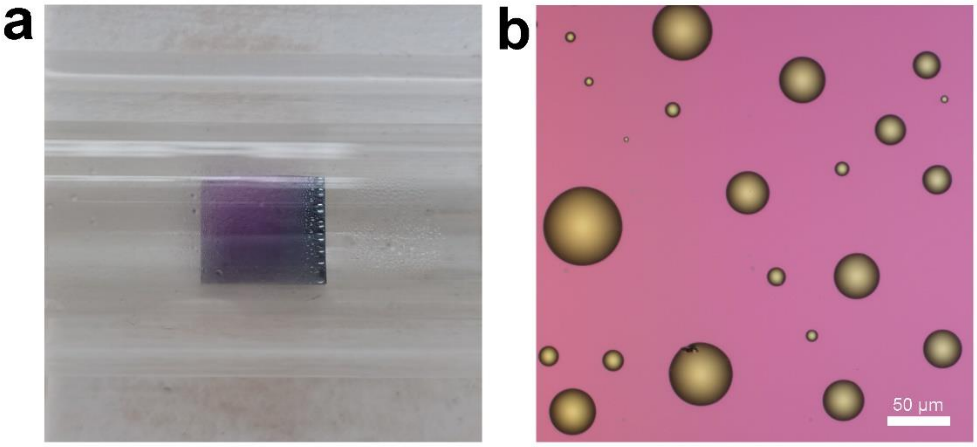

Supplementary Figure 5 | Self-condensation of benzimidazole with sublimation of precursor. (**a**) Self-condensed liquid droplets of benzimidazole appearing on the inner wall of the quartz tube (the CVD system was heated for about 15min). (**b**) Optical image of self-condensed liquid droplets of benzimidazole after solidification on the SiO_2/Si substrate.

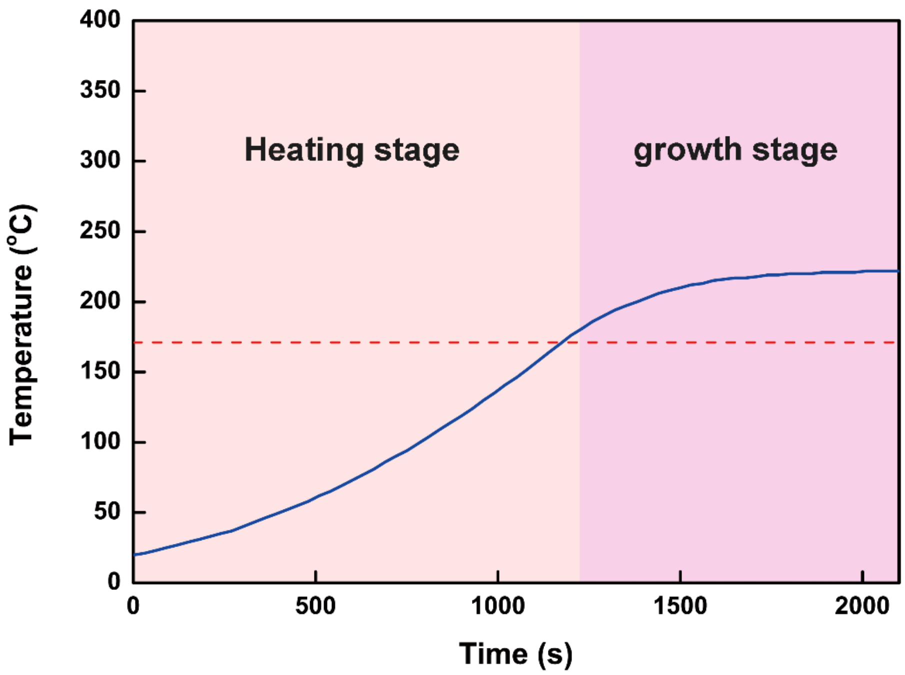

Supplementary Figure 6. Temperature change of the substrate during the SCA-CVD process. The blue solid line shows the temperature curve of the substrate, and the red dashed line represents the melting point (171 °C) of benzimidazole. Source data are provided as a Source Data file.

a

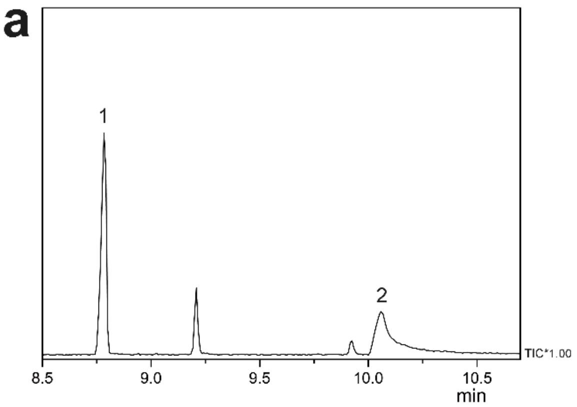

Peak Report TIC

Peak#	R.Time	I.Time	F.Time	Area	Area%
1	8.784	8.742	8.817	523070	59.44
2	10.059	10.000	10.267	356870	40.56

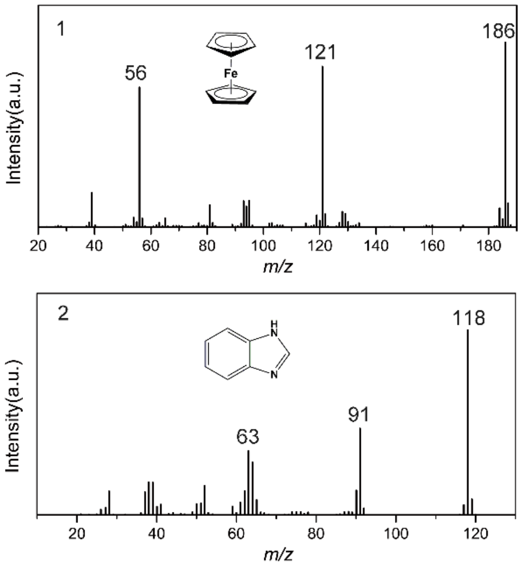

b

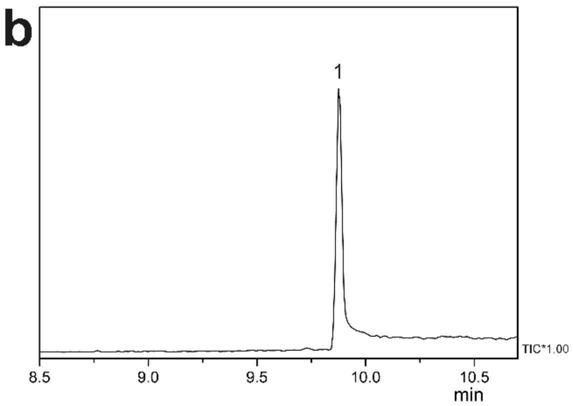

Peak Report TIC

Peak#	R.Time	I.Time	F.Time	Area	Area%
1	9.878	9.825	10.033	367841	100.00

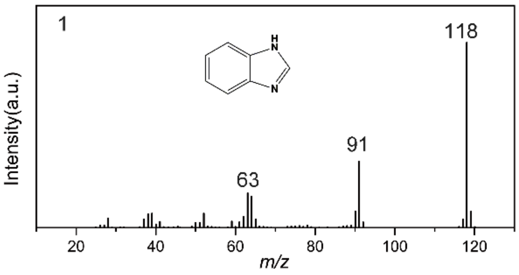

c

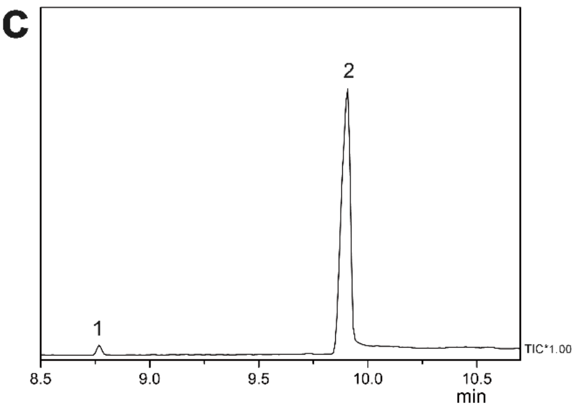

Peak Report TIC

Peak#	R.Time	I.Time	F.Time	Area	Area%
1	8.768	8.733	8.800	23040	1.97
2	9.906	9.833	10.017	1144293	98.03

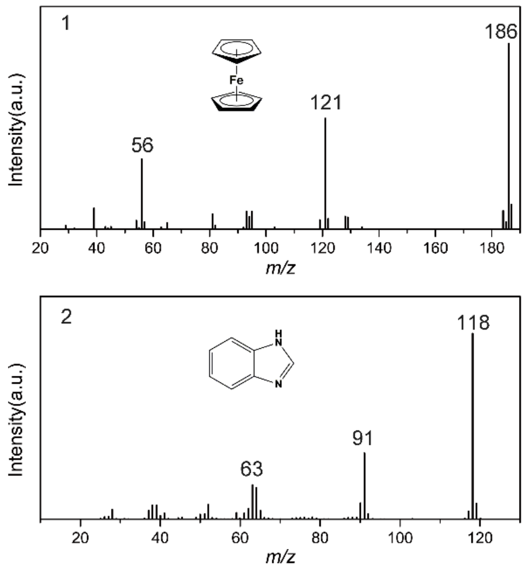

d

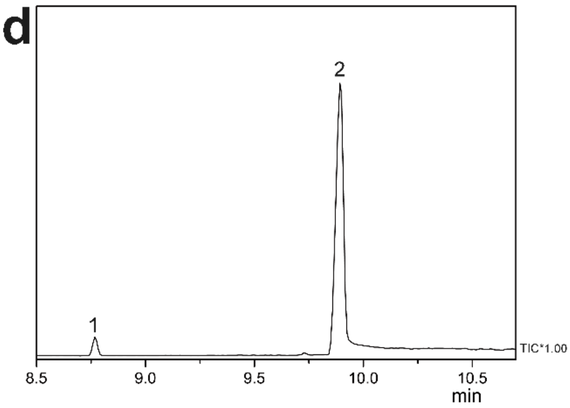

Peak Report TIC

Peak#	R.Time	I.Time	F.Time	Area	Area%
1	8.768	8.725	8.808	32130	3.55
2	9.894	9.825	10.108	872038	96.45

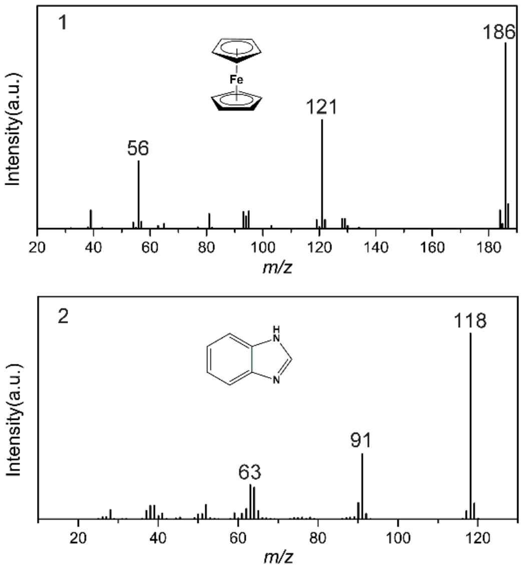

e

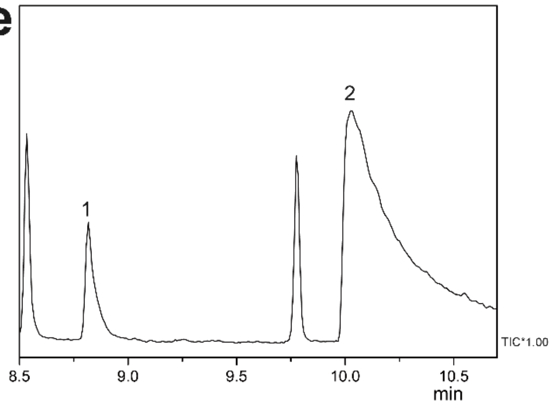

Peak Report TIC

Peak#	R.Time	I.Time	F.Time	Area	Area%
1	8.817	8.775	8.950	83344	8.33
2	10.029	9.958	10.650	916806	91.67

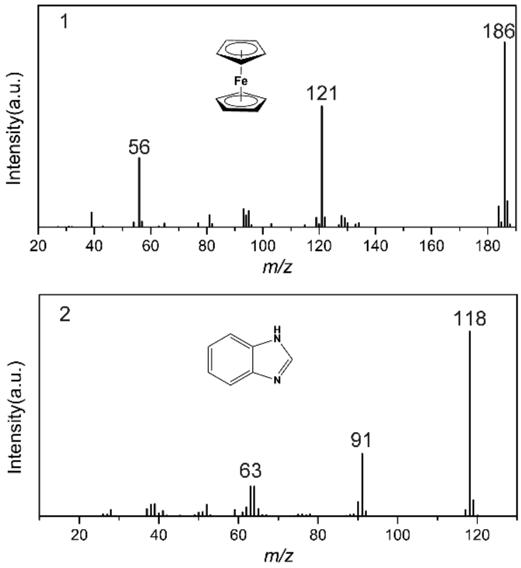

f

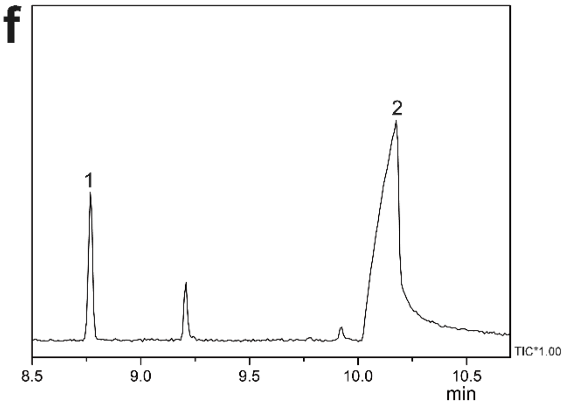

Peak Report TIC

Peak#	R.Time	I.Time	F.Time	Area	Area%
1	8.769	8.733	8.800	150908	11.02
2	10.174	10.008	10.392	1218973	88.98

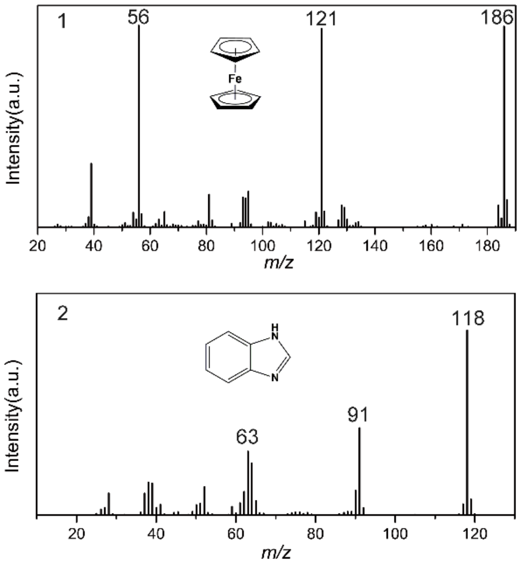

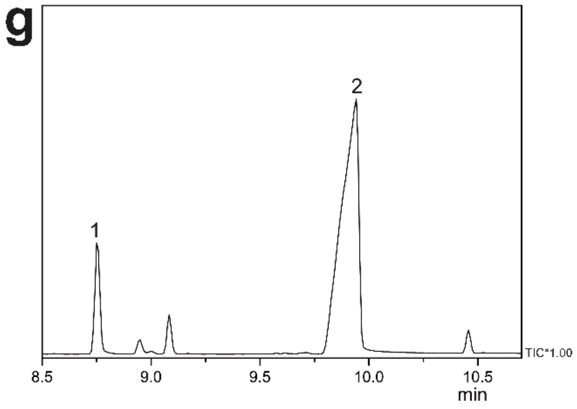

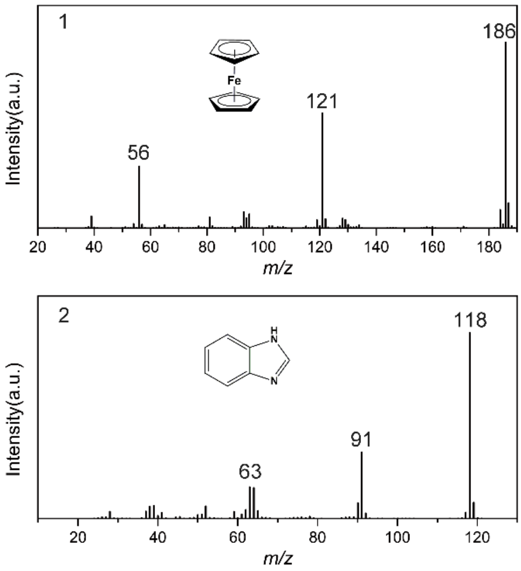

Peak Report TIC

Peak#	R.Time	I.Time	F.Time	Area	Area%
1	8.753	8.717	8.850	1610742	11.86
2	9.939	9.783	10.075	11968653	88.14

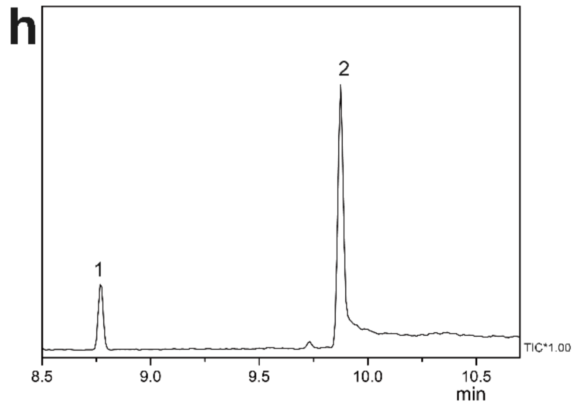

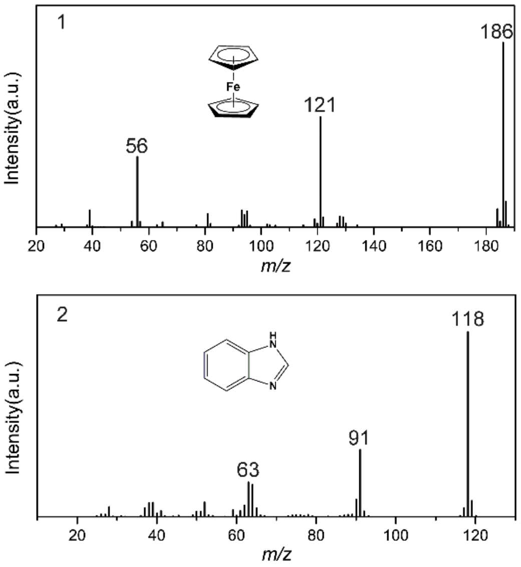

Peak Report TIC

Peak#	R.Time	I.Time	F.Time	Area	Area%
1	8.770	8.725	8.817	52868	14.29
2	9.875	9.833	10.050	316969	85.71

Supplementary Figure 7. (**a**) The GC-MS spectra of the reference sample. A mixture of benzimidazole and ferrocene with a molar ratio of 1:1 was used as a reference sample for the GC-MS test. The GC-MS spectra of the droplets on SiO_2/Si substrate when heated for 14 min (**b**), 15 min (**c**), 16 min (**d**), 18 min (**e**), 20 min (**f**), 25 min (**g**) and 30 min (**h**). The results show that the droplet component ranges from pure benzimidazole to a mixture of benzimidazole and ferrocene. Source data are provided as a Source Data file.

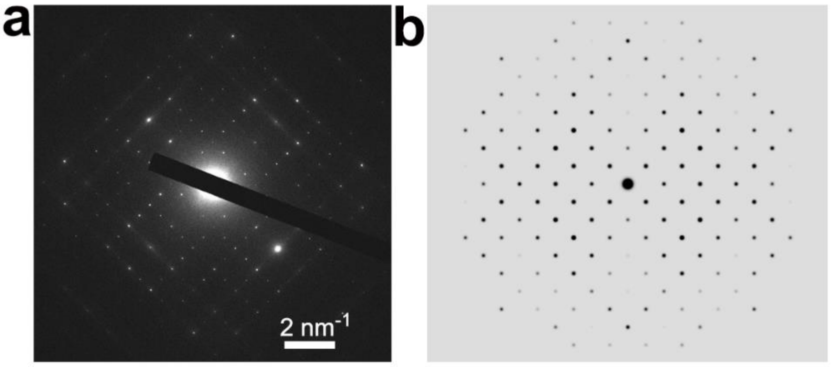

Supplementary Figure 8. (**a**) The experimental SAED pattern of a $Fe_n(bim)_{2n}$ flake. (**b**) A simulated SAED pattern of $Fe_n(bim)_{2n}$ flake down the *c* axis.

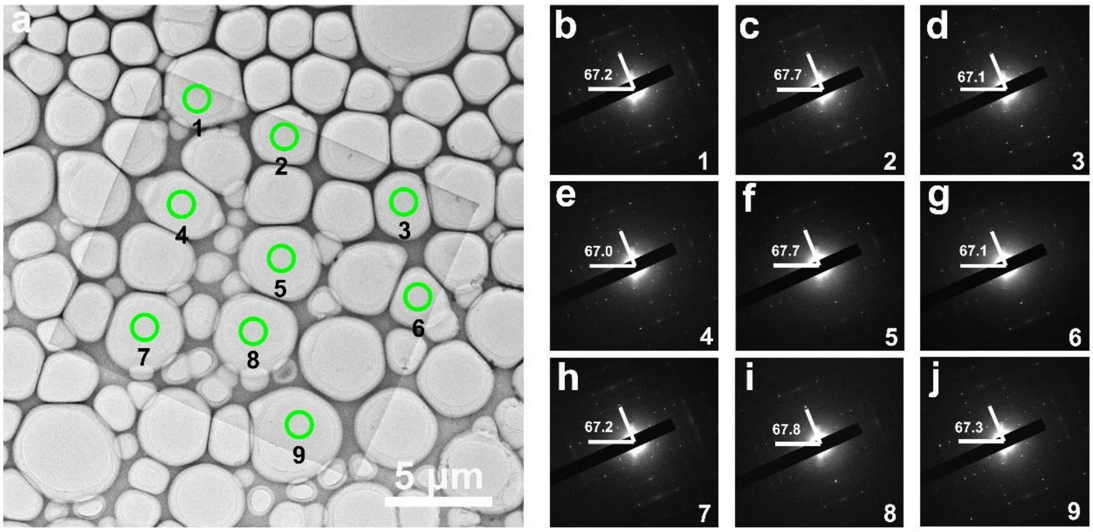

Supplementary Figure 9. (**a**) Low-magnification TEM image of an individual $Fe_n(bim)_{2n}$ flake. (**b-j**) SAED patterns of nine domains enclosed by green circles in **a**. The distance between adjacent domains is measured to be 3.7~ 4.4 μm. Only one set of quasi-four-fold symmetric diffraction spots was observed in each area, indicating that these areas are single crystals. The lattice orientations extracted from the SAED patterns in the nine areas were found almost identical. The above results confirm that the entire rectangle flake of $Fe_n(bim)_{2n}$ is a single crystal.

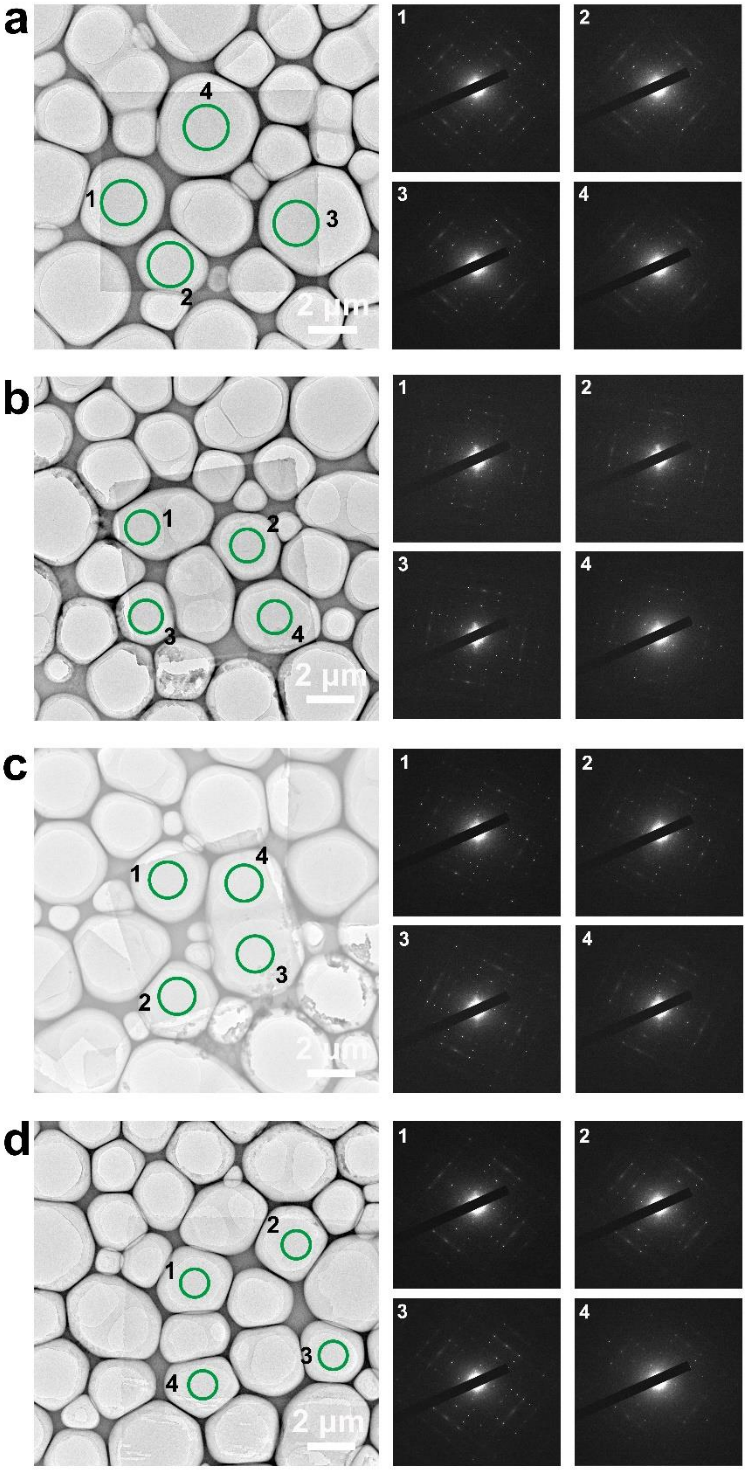

Supplementary Figure 10 | TEM characterizations of $Fe_n(bim)_{2n}$ flakes grown on different substrates. TEM images and SAED patterns at four randomly selected areas (green circles) of $Fe_n(bim)_{2n}$ flakes grown on SiO_2/Si (**a**), sapphire (**b**), quartz (**c**) and SiN (**d**). One set of diffraction spots and the same lattice orientation were demonstrated in different regions of the same rectangular flake, indicating all these individual flakes are single crystals. Therefore, the individual rectangular flakes of $Fe_n(bim)_{2n}$ grown on different substrates are all single crystals.

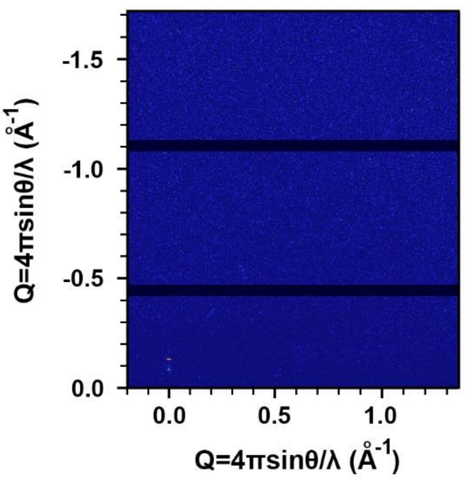

Supplementary Figure 11. The grazing incidence wide angle X-ray scattering (GIWAXS) image of $Fe_n(bim)_{2n}$ nanosheets. The GIWAXS characterization was carried out using XEUSS 2.0, but no signals have been detected on the $Fe_n(bim)_{2n}$ flakes. One of the main reasons for this result may be the energy of the light source used in the GIWAXS measurement is low for atomically thin 2D materials to produce detectable signals.

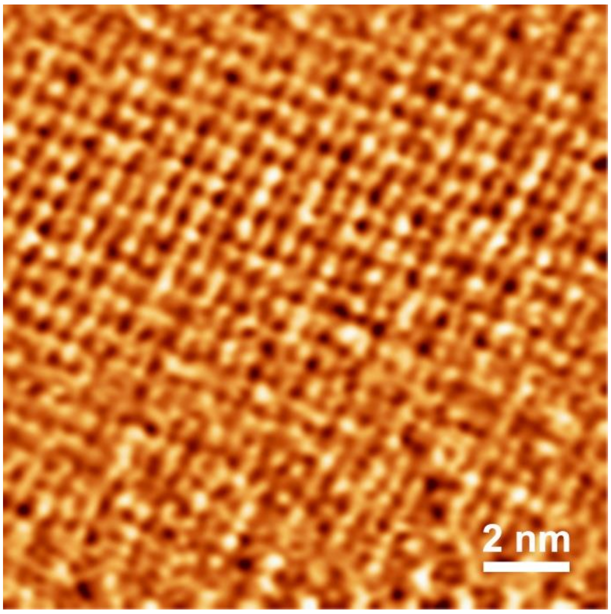

Supplementary Figure 12. HRAFM image of $Fe_n(bim)_{2n}$ after FFT processing of Fig. 3c.

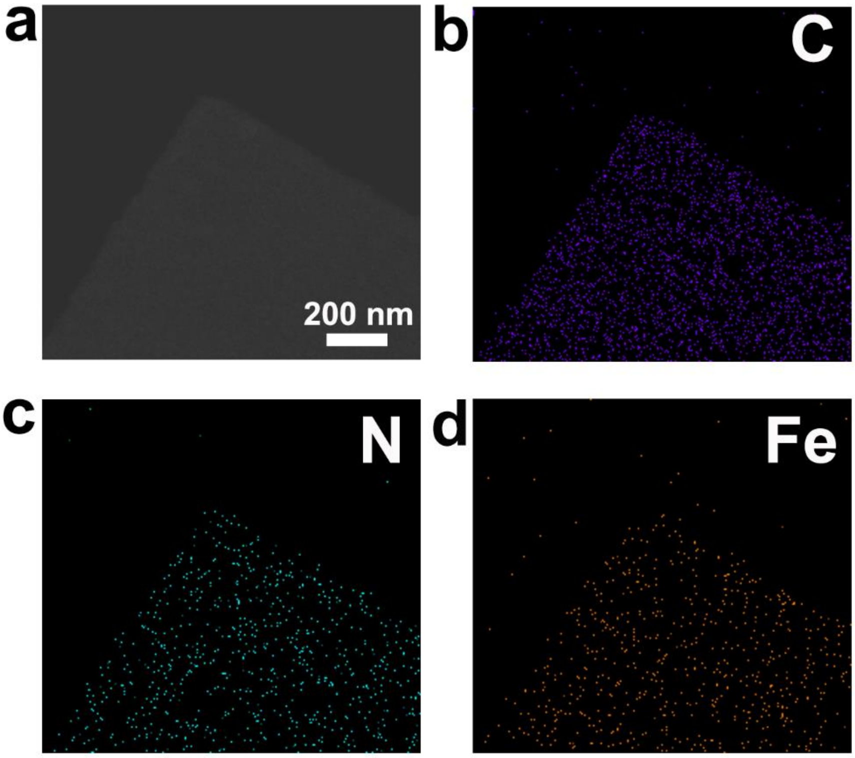

Supplementary Figure 13 | STEM-EDS mapping of $Fe_n(bim)_{2n}$ flake. STEM image of a $Fe_n(bim)_{2n}$ flake (**a**) and corresponding elemental maps for C (**b**), N (**c**) and Fe (**d**). STEM-EDS mapping was obtained by a JEOL JEM-F200 with acceleration voltage 200 kV.

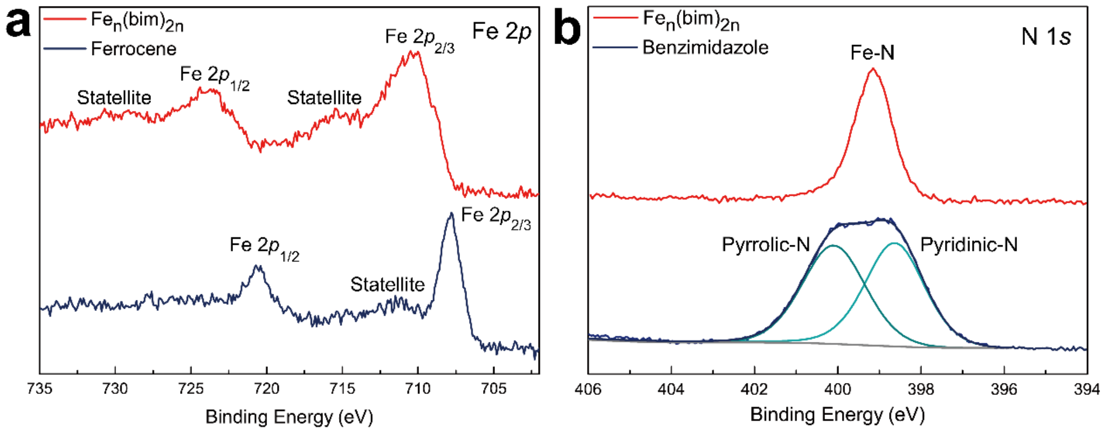

Supplementary Figure 14. (**a**) Fe 2*p* XPS spectra of $Fe_n(bim)_{2n}$ and ferrocene. (**b**) N 1*s* XPS spectra of $Fe_n(bim)_{2n}$ and benzimidazole. XPS spectra were obtained using ESCALab250Xi. In the Fe 2*p* spectrum of ferrocene, the peaks at 707.8 and 720.6 eV are assigned to the binding energies of the $2p_{3/2}$ and $2p_{1/2}$ orbitals of Fe(II) species, respectively. A weak peak at 711.3 eV can be attributed to a satellite peak, an indicator of Fe(II) in low-spin configuration. The $2p_{3/2}$ and $2p_{1/2}$ orbitals of Fe(II) in the $Fe_n(bim)_{2n}$ were observed at 710.5 and 723.9 eV respectively, shifting toward higher binding energies than those in the ferrocene. This can be explained by the decrease in electron cloud density around Fe atoms in $Fe_n(bim)_{2n}$ compared to ferrocene, as benzimidazole has stronger electron-withdrawing ability than the cyclopentadienyl group of ferrocene. The satellite peaks for $Fe_n(bim)_{2n}$ appear at 715.0 and 729.2 eV, suggesting the Fe(II) is in high-spin configuration[1]. The N 1*s* of benzimidazole can be divided into two peaks at 398.6 and 400.1 eV, which can be assigned to pyridinic N and pyrrolic N on the basis of the respective binding energies. Only one peak in the N 1*s* spectrum of $Fe_n(bim)_{2n}$ was observed at 399.1 eV. This change can be attributed to the coordination of two nitrogen atoms of benzimidazole with Fe (II) atoms to form Fe-N bonds. (The intensity normalization of XPS spectra was carried out.) Source data are provided as a Source Data file.

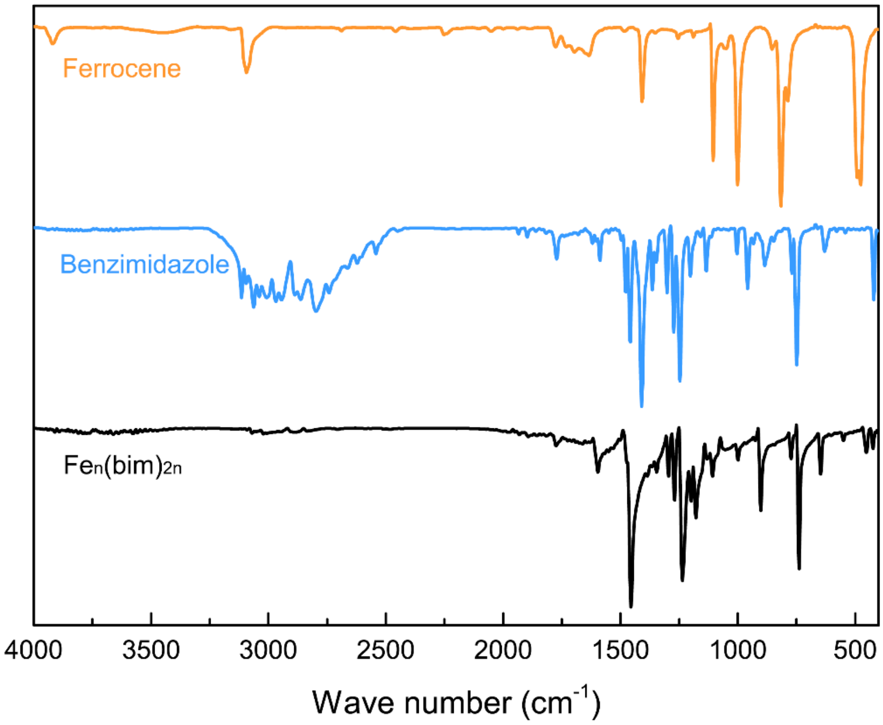

Supplementary Figure 15. FT-IR spectra of $Fe_n(bim)_{2n}$, ferrocene and benzimidazole. FT-IR spectroscopy was recorded on VERTEX 70v. The characteristic bands of 3100-2600 cm^{-1} assigned to N-H vibrations[2] in benzimidazole disappears in the spectrum of $Fe_n(bim)_{2n}$. It indicates the substitution of N-H in benzimidazole and formation of coordination bonds between Fe and N. (The intensity normalization of infrared spectra was carried out.) Source data are provided as a Source Data file.

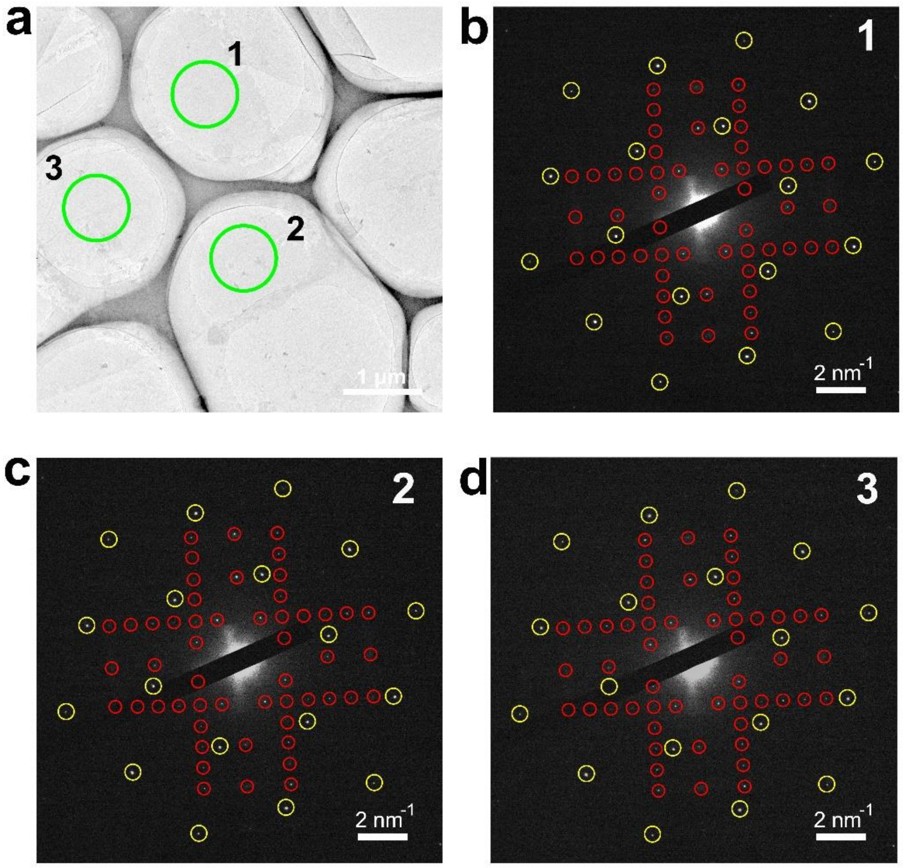

Supplementary Figure 16 | TEM characterizations of $Fe_n(bim)_{2n}/MoS_2$ heterostructures. (**a**) Low-magnification TEM image of $Fe_n(bim)_{2n}/MoS_2$ heterostructure. (**b-d**) SAED patterns for three regions indicated by green circles. The three regions all demonstrated two different sets of diffraction patterns, one set of six-fold diffraction patterns (yellow circle) belonging to MoS_2 and one set of quasi-four-fold diffraction patterns (red circle) belonging to $Fe_n(bim)_{2n}$. This suggests all the three regions are composed of single-crystalline $Fe_n(bim)_{2n}$ stacked onto the single-crystalline MoS_2. Comparing the quasi-four-fold diffraction patterns of the three regions, the same direction of arranged diffraction spots was found, which suggests $Fe_n(bim)_{2n}$ in the three regions share the same lattice orientation. Therefore, the entire $Fe_n(bim)_{2n}$ flake on MoS_2 is single-crystal.

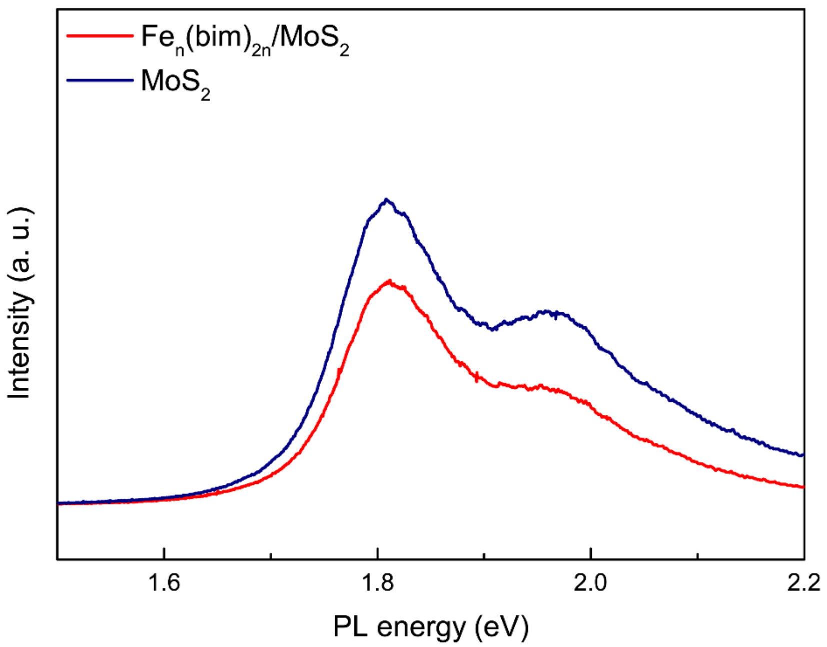

Supplementary Figure 17. Photoluminescence spectra of $Fe_n(bim)_{2n}/MoS_2$ heterostructure (red line) and bare monolayer MoS_2 (blue line). Compared to MoS_2, no apparent photoluminescence shift and quenching occurred in the heterostructure. This suggests no or very weak energy/charged carrier transfer between $Fe_n(bim)_{2n}$ and MoS_2 in the heterostructure. Source data are provided as a Source Data file.

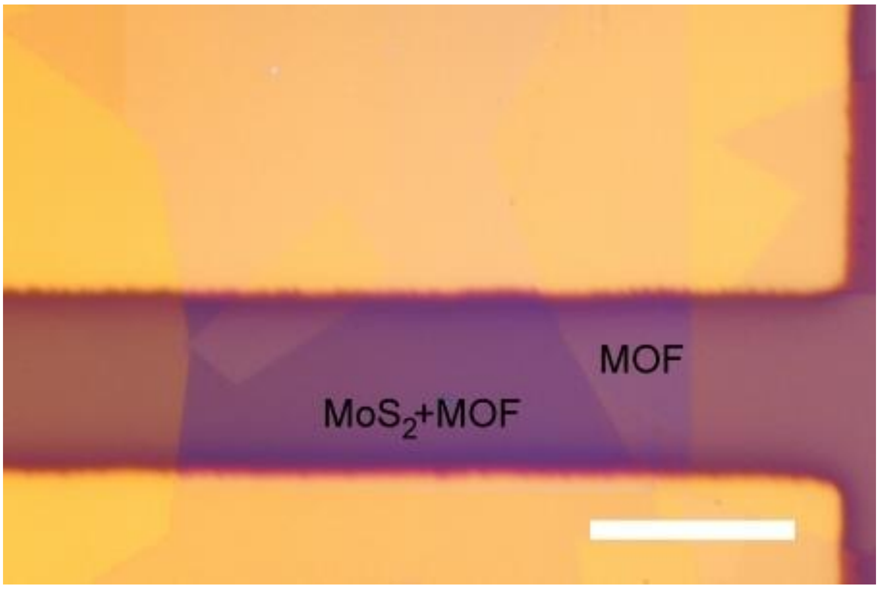

Supplementary Figure 18. The optical image of the $Fe_n(bim)_{2n}/MoS_2$ sensor. Scale bars, 20 μm.

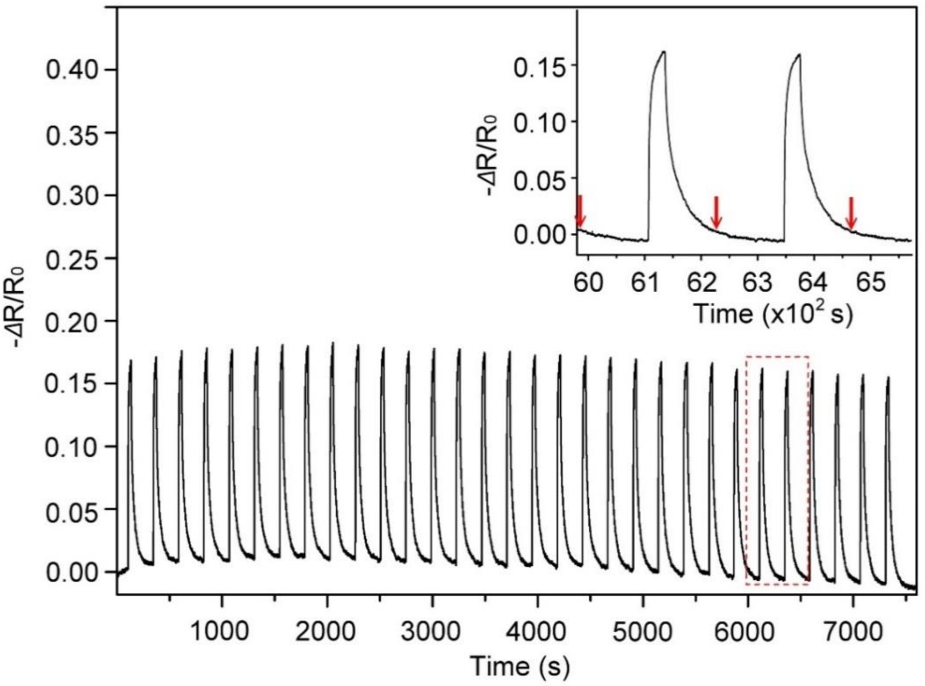

Supplementary Figure 19. Response of the $Fe_n(bim)_{2n}/MoS_2$ sensor during 30 successive cycles of exposure to NH_3 (20 ppm) and tert-butylamine (20 ppm) alternately. The red arrow represents the injection of tert-butylamine vapour. Source data are provided as a Source Data file.

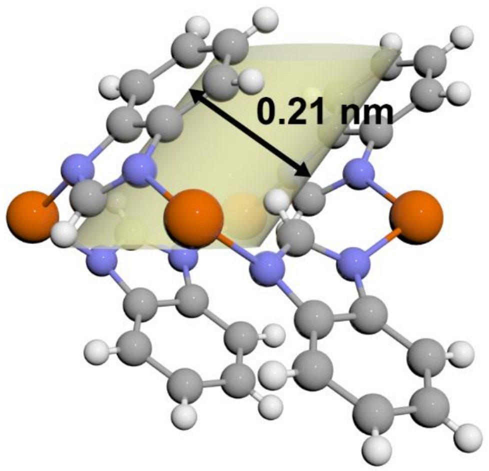

Supplementary Figure 20. 3D crystalline structure showing the pore of the $Fe_n(bim)_{2n}$ unit. Fe, N, C and H atoms are shown in orange, purple, gray and white balls, respectively.

Supplementary Table 1. Summary and comparison of NH_3 sensing performance obtained with different materials.

Structure	Sensing environment	Response/ Recovery time (s)	LOD[a] (ppm)	Linearity range (ppm)	Amine selectivity	Ref.
Mesoporous carbons	RT[b]	120/240	1	—	NH_3, benzene, etc.	[3]
S-SWNT	RT	60-600/—	—	—	NH_3, NO_2	[4]
Graphene aerogel	RT	100/500	0.01	0.02-85	NH_3, toluene, etc.	[5]
RGO	RT	~300-800/ ~1000-3500	—	200-2800	NH_3, methanol, etc.	[6]
Polyaniline-TiO_2	RT	~35-45/ ~140-155	—	—	—	[7]
Ag	RT	180-600/—	—	1-10	—	[8]
CuO-MnO_2	RT	120/600	—	—	—	[9]
ZnO Films	RT	~92–110/ ~111–113	5	—	NH_3, methanol, etc.	[10]
Silica modified CeO_2	RT	32-760/ 141-2800	0.5	0.5-80	NH_3, H_2 etc.	[11]
SnS_2	RT	300/—	0.02	—	NH_3, H_2O_2, etc.	[12]
DNTT	RT	95/—	0.01	0.01-1	NH_3, H_2O_2, etc.	[13]
Cu_3HHTP_2	RT	81.6/546.6	0.5	1-100	NH_3, CO, etc.	[14]
Cu_3(HHTP)(THQ)	RT	99/154.2	0.02–0.35	1-~$5x10^5$	NH_3, ethanol, etc.	[15]
$Ti_3C_2T_x$ film	RT	—	$0.13x10^{-3}$	—	NH_3, ethanol, etc.	[16]
WS_2 nanoflakes	RT	~120/~150	—	1-5	NH_3, benzene, etc.	[17]
Few layered n-typed $MoTe_2$	RT	—	~1	2-10	NH_3, NO_2	[18]
Film of MoS_2 powder	150 °C	400/~2200	—	10-50	NH_3, H_2, etc.	[19]
Au-dope MoS_2 nanoflakes	90 °C	306/367	10	10-200	NH_3, toluene, etc.	[20]
Monolayer MoS_2	RT	—	2.5	—	—	[21]
$Fe_n(bim)_{2n}/MoS_2$	RT	9-60/80-200	0.5	1-100	NH_3, amines	**This work**

[a] LOD, limit of detection, [b] RT, room temperature.

Supplementary References

1. López-Cabrelles, J. et al. Isoreticular two-dimensional magnetic coordination polymers prepared through pre-synthetic ligand functionalization. *Nat. Chem.* **10,** 1001–1007 (2018).
2. Nesmeyanov, A. N. et al. Iron cyclopentadienylcarbonyl complexes with azoles. I. Complexes of imidazoles and vicinal triazoles. *Inorg. Chim. Acta* **23,** 155-162 (1977).
3. Luo, W. et al. A micelle fusion-aggregation assembly approach to mesoporous carbon materials with rich active sites for ultrasensitive ammonia sensing. *J. Am. Chem. Soc.* **138,** 12586−1259 (2016).
4. Kong, J. et al. Nanotube molecular wires as chemical sensors. *Science* **287,** 622–625 (2000).
5. Alizadeh, T. & Ahmadian, F. Thiourea-treated graphene aerogel as a highly selective gas sensor for sensing of trace level of ammonia. *Anal. Chim. Acta* **897,** 87−95 (2015).
6. Ghosh, R., Midya, A., Santra, S., Ray, S. K. & Guha, P. K. Chemically reduced graphene oxide for ammonia detection at room temperature. *ACS Appl. Mater. Interfaces* **5,** 7599–7603 (2013).
7. Bairi, V. G. et al. Ammonia gas sensing behavior of tanninsulfonic acid doped polyaniline-TiO_2 composite. *Sensors* **15,** 26415−26429; (2015).
8. Athawale, A. A. & Katre, P. P. Ag dispersed conducting polyaniline nanocomposite as a selective sensor for ammonia. *J. Metastable Nanocryst. Mater.* **23,** 323−326 (2005).
9. Bhuvaneshwari; S., Papachan; S. & Gopalakrishnan N. Free standing CuO-MnO_2 nanocomposite for room temperature ammonia sensing. *AIP Conf. Proc.* **1832,** 050126 (2017).
10. Ponnusamy, D. & Madanagurusamy, S. Nanostructured ZnO films for room temperature ammonia sensing. *J. Electron. Mater.* **43,** 3211–3216 (2014).
11. Wang, J. et al. Enhanced NH_3 gas-sensing performance of silica modified CeO_2 nanostructure based sensors. *Sens. Actuators, B* **255,** 862–870 (2018).
12. Chen, H. et al. Suspended SnS_2 layers by light assistance for ultrasensitive ammonia detection at room temperature. *Adv. Funct. Mater.* **28,** 1801035 (2018).
13. Lu, J. et al. Porous organic field-effect transistors for enhanced chemical sensing performances. *Adv. Funct. Mater.* **27,** 1700018 (2017).
14. Yao, M-S. et al. Layer-by-layer assembled conductive metal–organic framework nanofilms for room-temperature chemiresistive sensing. *Angew. Chem. Int. Ed.* **56,** 16510–16514 (2017).
15. Yao, M-S. et al. A dual-ligand porous coordination polymer chemiresistor with modulated conductivity and porosity. *Angew. Chem. Int. Ed.* **59,** 172–176 (2020).
16. Kim, S. J. et al. Metallic $Ti_3C_2T_x$ MXene gas sensors with ultrahigh signal-to-noise ratio. *ACS Nano* **12,** 986–993 (2018).
17. Li, X. et al. WS_2 nanoflakes based selective ammonia sensors at room temperature. *Sens. Actuators, B* **240,** 273–277, (2017).
18. Feng, Z. et al. Highly sensitive $MoTe_2$ chemical sensor with fast recovery rate through gate biasing. *2D Mater.* **4,** 025018 (2017).
19. Kim, T. et al. Drastic gas sensing selectivity in 2-dimensional MoS_2 nanoflakes by noble metal decoration. *ACS Nano* **17,** 4404−4413 (2023).
20. Burman, D., Raha, H., Manna, B., Pramanik, P. & Guha, P. K. Substitutional doping of MoS_2 for superior gas-sensing applications: a proof of concept. *ACS Sens.* **6,** 3398–3408 (2021).
21. Park, J., Mun, J., Shin, J-S. & Kang, S-W. Highly sensitive two-dimensional MoS_2 gas sensor decorated with Pt nanoparticles. *R. Soc. Open Sci.* **5,** 181462 (2018).
